# Tissue-Type Plasminogen Activator-Inhibitor Complex as an Early Predictor of Septic Shock: A Retrospective, Single-Center Study

**DOI:** 10.1155/2022/9364037

**Published:** 2022-04-04

**Authors:** Lincui Zhong, Jianlin Dou, Qingwei Lin, Longping He, Qingbo Zeng, Jingchun Song

**Affiliations:** ^1^Department of Critical Care Medicine, The 908th Hospital of Chinese Logistical Support Force, Nanchang 330002, China; ^2^Nanchang Key Laboratory of Thrombosis and Hemostasis, Nanchang 330002, China; ^3^Intensive Care Unit, Nanchang Hongdou Hospital of TCM, Nanchang, 330002 Jiangxi Province, China

## Abstract

**Background:**

Sepsis can progress to septic shock and death, and identifying biomarkers of this progression may permit timely intervention to prevent it. This study explored whether levels of tissue-type plasminogen activator-inhibitor complex (t-PAIC) in serum can predict septic shock early.

**Methods:**

We retrospectively analyzed 311 sepsis patients who had been admitted to the intensive care unit (ICU) at our tertiary care hospital between May 2018 and April 2021, and we divided them into those who progressed to septic shock (*n* = 203) or not (*n* = 108) based on sepsis-3 definition. After matching patients in the two groups based on propensity scoring, we screened for risk factors of septic shock using logistic regression. We assessed potential predictors of such shock based on the area under the receiver-operating characteristic curve (AUC), Kaplan-Meier survival curves, and correlation analysis.

**Results:**

After propensity score matching to generate two equal groups of 108 patients, we found that serum t-PAIC was significantly higher in septic shock patients. Uni- and multivariate logistic regression identified t-PAIC as an independent risk factor for septic shock (OR 1.14, 95% CI 1.09–1.19, *P* < 0.001) and a biomarker that predicted it with an AUC up to 0.875 (95% CI, 0.829-0.920). Based on the optimal cut-off of t‐PAIC = 17.9 ng/mL, we found that patients at or above this threshold had significantly higher lactate levels and scores on the Acute Physiology and Chronic Health Evaluation II (APACHE II) and Sequential Organ Failure Assessment (SOFA). Such patients also had significantly worse survival (HR 2.4, 95% CI 1.38–4.34, *P* = 0.004). Spearman's correlation coefficients were 0.66 between t-PAIC and lactate, and 0.52 between t-PAIC and SOFA.

**Conclusions:**

Serum levels of t-PAIC may be an independent risk factor for septic shock, and they may correlate with the severity of such shock.

## 1. Background

Sepsis is a complex syndrome in which the body's unbalanced response to infection can cause life-threatening organ dysfunction, leading to high morbidity and mortality [1, 2]. Sepsis can progress to septic shock, which involves a combination of severe circulatory, cellular, and metabolic disorders [3, 4]. In China, this progression is associated with an increase in the 90-day mortality rate from 2.78% to 51.94% [5]. Therefore, early recognition of this progression may allow timelier, more effective intervention [6].

Sepsis begins when infecting pathogens release endotoxins that injure vascular endothelial cells [7], and this injury induces the release of tissue factors, activation of the coagulation pathway, microthrombus formation, and tissue ischemia, ultimately leading to organ dysfunction [8]. Endothelial cells can also secrete tissue-type plasminogen activator (t-PA), which degrade microthrombi, as well as plasminogen activator inhibitor-1 (PAI-1), which inhibits t-PA [9]. Levels of the complex between t-PA and PAI-1, called t-PAIC, therefore reflect the severity of endothelial cell damage and the resulting level of fibrinolysis [10]. Sepsis can then progress to septic shock when injured vascular endothelial cells release abundant cytokines that induce a systemic inflammatory response [11, 12].

The present study examined whether levels of t-PAIC could serve as an early-warning indicator of septic shock.

## 2. Patients and Methods

### 2.1. Study Design and Patients

This retrospective study enrolled sepsis patients who had been admitted to the ICU of our tertiary hospital (Nanchang, China) from May 2018 to April 2021. To be enrolled, patients had to have suspected or confirmed infection and a score of at least 2 points on the SOFA [1]. Patients were excluded if they were younger than 16 years, if they had “do not resuscitate” status, or if they had chronic insufficiency of the liver or kidneys.

Sepsis patients were further diagnosed with septic shock if they had persistent hypotension and lactate levels > 2 mmol/L despite adequate volume therapy and if they required vasopressors in order to maintain a mean arterial pressure ≥ 65 mmHg [2].

This study was approved by the Ethics Committee of our hospital, which waived the requirement for informed consent because the patients or their legal guardians, at admission, gave written consent for their anonymized medical records to be published for research purposes.

### 2.2. Data Collection

Demographic and clinical data including age, sex, source of infection, and clinical outcome were retrieved from electronic medical records. The following analyses were performed within 2 h of admission to the ICU: white blood cell count, platelet count, activated partial thromboplastin time (APTT), prothrombin time (PT), thrombin time (TT), as well as levels of C-reactive protein (CRP), fibrinogen, fibrin degradation products (FDP), D-dimer, antithrombin III (ATIII), lactate, thrombin-antithrombin complex (TAT), thrombomodulin (TM), *α*2-plasmin inhibitor-plasmin complex (PIC), and t-PAIC. Scores on the SOFA [13] and disseminated intravascular coagulation (DIC) [14] scales were calculated in the ICU upon initial diagnosis of sepsis. Scores on the APACHE II scale [15] were calculated based on the worst parameters during the first 24 h in the ICU.

### 2.3. Statistical Analysis

All statistical analyses were performed using SPSS software (version 26.0; IBM, Chicago, IL, USA) and GraphPad Prism (version 8.0; GraphPad Software Inc, La Jolla, California). All statistical analyses were two-tailed, and differences were considered significant if associated with *P* < 0.05.

All continuous data were tested for normality. Normally distributed data were expressed as mean ± standard deviation, and intergroup differences in such data were assessed for significance using Student's *t* test. Skewed data were expressed as median (interquartile range), and intergroup differences were assessed using the Mann–Whitney *U* test. Categorical data were expressed as numbers (percentages), and intergroup differences were assessed using the *χ*^2^ test.

Given the observational study design, we reduced potential confounding from baseline differences by matching the patients in different groups 1 : 1 based on propensity scores calculated using maximize execution performance and a fixed caliper width of 0.2. Uni- and multivariate logistic regression was performed on the matched patient groups in order to identify risk factors for septic shock based on odds ratios (ORs) and the corresponding 95% confidence intervals (CIs). All variables that were associated with *P* < 0.05 in the univariate analysis were included in the multivariate model. Independent risk factors were those that emerged as statistically significant in both uni- and multivariate regression.

Potential biomarkers of septic shock were assessed in terms of the AUC. Survival rates between groups were compared using the Kaplan-Meier method and log-rank test. Spearman's rank correlation was used to assess associations of serum t-PAIC with lactate levels and SOFA scores.

## 3. Results

### 3.1. Baseline Characteristics of Patients

**Figure 1:**
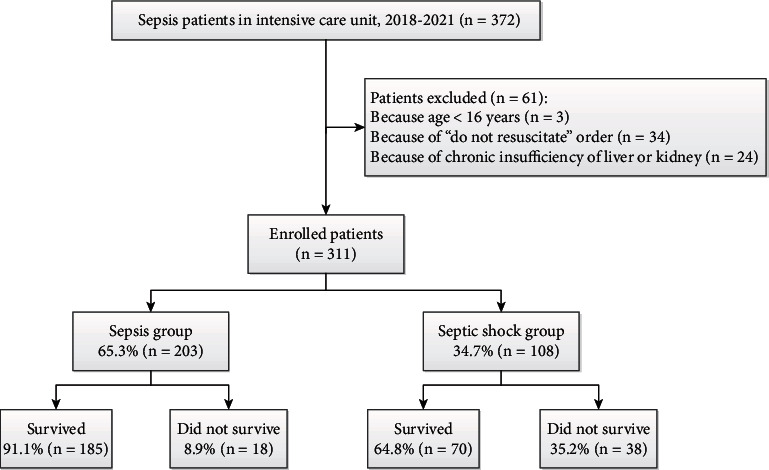
Patient flow diagram.

Between May 2018 and April 2021, 372 patients with sepsis were treated at our hospital. We excluded 3 patients younger than 16 years, 34 patients with “do not resuscitate” orders, and 24 patients with chronic insufficiency of the liver or kidneys. The remaining 311 patients were enrolled in our study, of whom 108 (34.7%) were diagnosed with septic shock (Figure 1). Of those diagnosed with septic shock, 38 (35.2%) died.

Without propensity score matching, patients in the sepsis group were younger than those in the septic shock group (66.0 vs. 74.5 yr, *P* = 0.016; Table 1). The two groups did not differ significantly in sex composition, length of stay in the ICU, site of infection, white blood cell count, or CRP level. In contrast, the two groups did differ significantly in several other inflammatory indicators: the septic shock group showed longer PT, APTT, and TT; smaller fibrinogen and ATIII levels; higher D-dimer and FDP levels; and lower platelet count. The septic shock group also showed significantly higher lactate level, mortality rate, and scores on the APACHE II, SOFA, and DIC.

After propensity score matching, there were no significant differences in general characteristics between the sepsis group and the septic shock group, including age, gender, stay in the ICU, and infection site (Table 2).

### 3.2. Independent Risk Factors for Septic Shock

Univariate analysis of the 216 propensity score-matched patients identified the following variables associated with septic shock: white blood cell count, TM, TAT, t-PAIC, PT, APTT, TT, fibrinogen, FDP, D-dimer, AT3, and platelet count (Table 2). However, only t-PAIC emerged from multivariate logistic analysis as an independent predictor of septic shock (OR 1.14, 95% CI 1.09–1.19, *P* < 0.001).

### 3.3. Ability of t-PAIC to Predict Septic Shock

**Figure 2:**
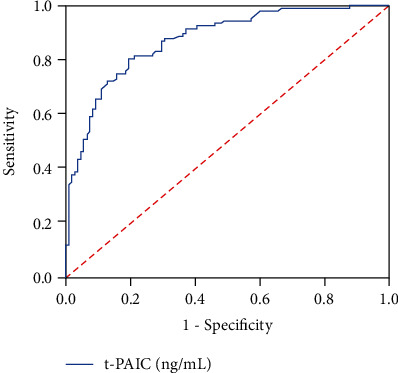
Receiver operating characteristic curve to assess the ability of t-PAIC to predict septic shock.

The AUC for t-PAIC to predict septic shock among the 216 propensity score-matched patients was 0.875 (95% CI: 0.829-0.920) for the optimal cut-off value of 17.9 ng/mL, which gave sensitivity and specificity of 0.806 (*P* < 0.001; Figure 2).

### 3.4. Differences in Sepsis Severity and Survival between Patients Stratified by t-PAIC

**Figure 3:**
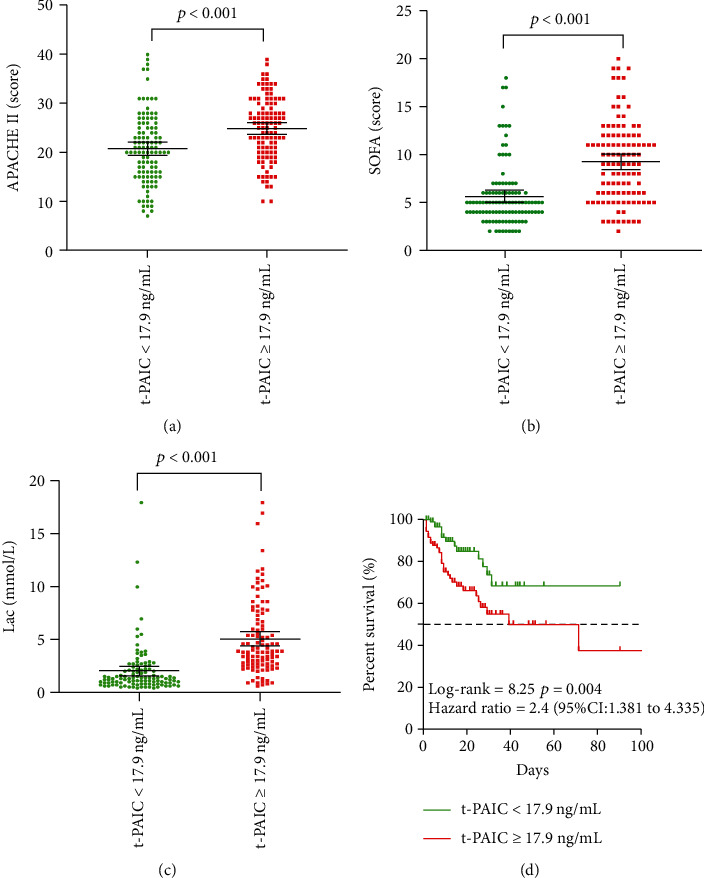
Comparison of propensity-score matched patients stratified based on optimal t-PAIC cut-off of 17.9 ng/mL. (a) Score on APACHE II. (b) Score on SOFA. (c) Lactate levels. (d) Kaplan-Meier survival curves.

Stratifying the 216 propensity score-matched patients based on the optimal t-PAIC cut-off of 17.9 ng/mL showed that those at or above this level had significantly higher APACHE II score, SOFA score, and lactate level (Figures 3(a)–3(c)), as well as significantly worse survival (Figure 3(d)).

### 3.5. Correlation Analysis

**Figure 4:**
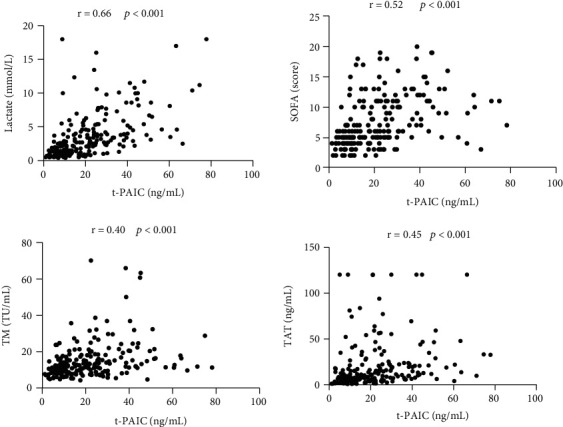
Analysis of correlations of t-PAIC with lactate, TM, TAT, or score on the SOFA.

Levels of t-PAIC in serum correlated strongly with lactate levels [correlation coefficient (*r*) = 0.66, *P* < 0.001], but moderately with SOFA score (*r* = 0.52, *P* < 0.001), TAT (*r* = 0.45, *P* < 0.001), and TM (*r* = 0.40, *P* < 0.001) (Figure 4). The other variables had weak correlations with t-PAIC levels including PT, APECHE II score, APTT, TT, d-dimer, and FDP.

## 4. Discussion

This retrospective, observational, single-center study provides the first evidence that t-PAIC may be a valuable biomarker for early prediction of septic shock. We measured significantly higher levels of t-PAIC in patients with septic shock than in those with sepsis, and the levels correlated positively with lactate levels and SOFA scores. Multivariate analysis identified t-PAIC levels ≥ 17.9 ng/mL as an independent risk factor for septic shock in sepsis patients (OR 1.14, 95% CI 1.09–1.19, *P* < 0.001). Patients above this threshold had significantly higher lactate levels and scores on the APACHE II and SOFA than patients below the threshold. Our results suggest that assaying t-PAIC in sepsis patients may help screen for those at high risk of such shock, enabling timely intervention that may mitigate its impact or even prevent it.

Septic shock substantially increases risk of mortality in sepsis patients [16], as we observed in our sample, where the mortality rate was 35.2% among those with septic shock and 8.9% among those with sepsis. In sepsis, endotoxins and inflammatory cytokines increase the permeability of the endothelial layer, leading to endothelial barrier dysfunction, vascular leakage, and reduced blood volume, which in turn reduce tissue and organ perfusion [17, 18]. Following endothelial cell injury, t-PA and PAI-1 are combined at 1 : 1 ratio to form the complex t-PAIC [19]. Our findings on the correlation between t-PAIC and risk of septic shock may help explain the previously reported association of t-PAIC with cardiovascular mortality in patients with heart failure and preserved ejection fraction [20]. In that study, t-PAIC did not correlate with New York Heart Association functional class or levels of the N-terminal prohormone from the brain natriuretic peptide. We propose that in the patients in that study, heart failure induced tissue hypoxia, which injured endothelial cells and reduced perfusion, triggering an increase in t-PAIC [21]. This may also explain why t-PAIC levels in our patients correlated obviously with the levels of TM and lactic acid [22, 23].

The t-PAIC levels can be used to assess fibrinolytic dysfunction [24], since they reflect the balance between fibrinolysis by t-PA [25] and inhibition of fibrinolysis by PAI-1 [26, 27]. Previous studies have reported that the levels of t-PAIC can be significantly increased in thrombosis patients suffered from liver disease [28, 29], malignant tumor [30], COVID-19 [31], acute myocardial infarction [32], or stroke [33]. Indeed, patients with heart failure and preserved ejection fraction in one study showed significantly higher t-PAIC levels if they had a history of stroke, diabetes, or arterial hypertension, all of which are associated with higher risk of thrombotic complications [20]. Our study proved that t-PAIC also increased significantly in patients with septic shock, but rising t-PAIC levels were caused by endothelial cell injury instead of hyperfibrinolysis. Actually, extensive microthrombosis resulted in multiple organ dysfunction in patients with septic shock due to fibrinolysis shutdown [34]. Levels of D-dimers and FDP have been proposed as risk factors for septic shock [35]. Those variables were strong univariate predictors of septic shock in our patients, but they were not significant in the multivariate model. Our results suggest that t-PAIC may be a more reliable biomarker of septic shock.

The mortality rate among our patients with septic shock (35.2%) was lower than the rate of 51.9% reported across 44 ICUs in China [5], even though our sample showed similar APACHE II and SOFA scores as the national sample. This discrepancy highlights the need to verify our single-center findings in larger, multisite studies. Future work should also continuously monitor relevant indices, in contrast to our analysis of data collected only within 2 h after admission to the ICU.

## 5. Conclusions

Our study provides the first evidence that t-PAIC levels may be an independent risk factor for septic shock, and they may correlate with the severity of such shock.

## Figures and Tables

**Table 1:** Clinic demographic characteristics of sepsis patients with or without septic shock, before and after propensity score matching.

Characteristics	Prematching			Postmatching		
	Sepsis group (*n* = 203)	Septic shock group (*n* = 108)	*P*	Sepsis group (*n* = 108)	Septic shock group (*n* = 108)	*P*
Age, yr	66.0 (52.0, 79.0)	74.5 (58.3, 84.0)	0.016	71.5 (55.3, 83.0)	74.5 (58.3, 84.0)	0.596
Male	123 (60)	64 (59.3)	0.899	59 (54.6)	64 (59.3)	0.492
ICU stay, d	9 (5, 20)	9 (5, 24)	0.95	8 (4, 20)	9 (5, 24)	0.462
Infection site						
Pulmonary	129 (63.5)	64 (59.3)	0.458	66 (61.1)	64 (59.3)	0.781
Abdominal	43 (21.2)	29 (26.9)	0.259	23 (21.3)	29 (26.9)	0.34
Genitourinary	11 (5.4)	4 (3.7)	0.502	4 (3.7)	4 (3.7)	1
Skin and soft tissue	10 (4.9)	3 (2.8)	0.546	9 (8.3)	3 (2.8)	0.075
Other	10 (4.9)	8 (7.4)	0.372	6 (5.6)	8 (7.4)	0.58
Inflammatory indicators						
White blood cell,10^9^/L	11.5 (8.2, 16.0)	13.3 (8.2, 19.8)	0.052	10.9 (7.7, 15.3)	13.3 (8.2, 19.8)	0.017
C-reactive protein, mg/L	57.5 (19.4, 131.8)	70.2 (25.0, 120.5)	0.458	55.6 (14.4, 122.4)	70.2 (25.0, 120.5)	0.224
Conventional coagulation tests						
PT, sec	14.1 (13.0, 15.8)	16.1 (14.1, 19.2)	<0.001	14.0 (12.9, 15.3)	16.1 (14.1, 19.2)	<0.001
APTT, sec	30.8 (27.4, 35.2)	34.0 (29.0, 42.1)	<0.001	30.6 (26.7, 34.4)	34.0 (29.0, 42.1)	<0.001
TT, sec	15.6 (14.4, 17.1)	16.7 (15.1, 18.5)	<0.001	15.3 (14.3, 16.6)	16.7 (15.1, 18.5)	<0.001
Fibrinogen,g/L	3.0 ± 1.0	2.7 ± 1.0	0.003	3.1 ± 1.0	2.7 ± 1.0	0.005
FDP, *μ*g/mL	6.60 (3.19, 15.89)	10.11 (4.53, 25.79)	0.008	5.65 (2.35, 12.63)	10.11 (4.53, 25.79)	0.001
D-dimer, *μ*g/mL	2.28 (0.88, 4.86)	3.45 (1.56, 7.21)	0.003	1.88 (0.74, 4.29)	3.45 (1.56, 7.21)	<0.001
AT3, %	75.0 (61.0, 93.0)	61.5 (46.0, 81.8)	<0.001	75.0 (58.0, 93.8)	61.5 (46.0, 81.8)	0.001
Platelets, 10^9^/L	178.0 (123.0, 244.0)	144.5 (70.3, 205.8)	0.001	183.0 (137.8, 248.8)	144.5 (70.3, 205.8)	<0.001
Coagulation biomarkers						
TM, TU/mL	11.0 (8.3, 15.3)	13.8 (11.0, 20.1)	<0.001	10.3 (7.9, 13.8)	13.8 (11.0, 20.1)	<0.001
TAT, ng/mL	7.4 (3.9, 15.2)	14.2 (7.0, 26.4)	<0.001	7.1 (3.9, 14.3)	14.2 (7.0, 26.4)	<0.001
PIC, *μ*g/mL	1.18 (0.70, 1.83)	1.07 (0.57, 2.44)	0.358	1.07 (0.65, 1.78)	1.07 (0.57, 2.44)	0.635
t-PAIC, ng/mL	10.9 (7.4, 17.0)	26.5 (19.7, 40.3)	<0.001	9.9 (6.8, 17.0)	26.5 (19.7, 40.3)	<0.001
Severity of sepsis						
APACHE II score	20.9 ± 6.3	26.3 ± 6.4	<0.001	19.4 ± 6.0	26.3 ± 6.4	<0.001
SOFA score	6.0 (4.0, 8.0)	10.5 (8.0, 13.0)	<0.001	5.0 (3.3, 5.0)	10.5 (8.0, 13.0)	<0.001
DIC score	1.0 (0, 3.0)	3.0 (1.0, 4.0)	<0.001	0.5 (0, 3.0)	3.0 (1.0, 4.0)	<0.001
Lactate, mmol/L	1.3 (0.9, 1.9)	4.3 (2.9, 7.5)	<0.001	1.1 (0.8, 1.7)	4.3 (2.9, 7.5)	<0.001
Deaths in ICU	18 (8.9)	38 (35.2)	<0.001	9 (8.3)	38 (35.2)	<0.001

Values are *n* (%), mean ± SD, or median (interquartile range), unless otherwise noted. APTT: activated partial thromboplastin time; AT: antithrombin; FDP: fibrin degradation product; PT: prothrombin time; PIC: *α*_2_-plasmin inhibitor-plasmin complex; TT: thrombin time; TM: thrombomodulin; TAT: thrombin-antithrombin complex; t-PAIC: tissue-type plasminogen activator-inhibitor complex.

**Table 2:** Uni- and multivariate analysis to identify risk factors for septic shock.

	Univariate		Multivariate	
Factor	Odds ratio (95% CI)	*P*	Odds ratio (95% CI)	*P*
White blood cell (×10^9^/L)	1.06 (1.02, 1.10)	0.003	—	—
TM (TU/mL)	1.10 (1.05, 1.15)	<0.001	—	—
TAT (ng/mL)	1.02 (1.00, 1.03)	0.016	—	—
t-PAIC (ng/mL)	1.16 (1.11, 1.21)	<0.001	1.14 (1.09, 1.19)	<0.001
PT (sec)	1.09 (1.01, 1.17)	0.037	—	—
APTT (sec)	1.06 (1.02, 1.09)	0.003	—	—
TT (sec)	1.18 (1.06, 1.32)	0.003	—	—
Fibrinogen (g/L)	0.67 (0.51, 0.89)	0.005	—	—
FDP (*μ*g/mL)	1.01 (1.00, 1.02)	0.034	—	—
D-dimer (*μ*g/mL)	1.09 (1.03, 1.16)	0.004	—	—
AT3 (%)	0.99 (0.98, 1.00)	0.007	—	—
Platelets (×10^9^/L)	1.00 (0.99, 1.00)	0.001	—	—

APTT: activated partial thromboplastin time; AT: antithrombin; CI: confidence interval; FDP: fibrin degradation product; PT: prothrombin time; TM: thrombomodulin; TAT: thrombin-antithrombin complex; t-PAIC: tissue-type plasminogen activator-inhibitor complex; TT: thrombin time.

## Data Availability

The datasets used and/or analyzed during the current study are available from the corresponding author on reasonable request.
